# Frequency of Vitamin a Deficiency in Children Hospitalized for Pneumonia: An Integrative Review

**DOI:** 10.3389/phrs.2022.1604500

**Published:** 2022-12-13

**Authors:** Amanda De Conceição Leão Mendes, Ana Mayara Gomes De Souza, Aryelly Dayane Da Silva Nunes, Javier Jerez-Roig, Isabelle Ribeiro Barbosa

**Affiliations:** ^1^ Department of Collective Health, Federal University of Rio Grande do Norte, Natal, Brazil; ^2^ University of Vic-Central University of Catalonia (UVIC-UCC), Barcelona, Spain

**Keywords:** children, review, pneumonia, hospitalization, vitamin A

## Abstract

**Objective:** To identify the frequency of vitamin A deficiency in children aged 6 months to 5 years hospitalized for pneumonia.

**Methods:** An integrative literature review was carried out, where searches were made by two independent researchers, with no language limits or publication time in the databases PubMed, LILACS, Web of Science, Scopus and CINAHL, and in the gray literature—OpenGrey, Proquest and Google Scholar. In the eligibility phase, the screened studies were read in full and those that did not answer the research question were excluded. Methodological quality was assessed using the Downs & Black (1998) checklist.

**Results:** 1642 articles were identified, after all stages of screening and selection, 10 studies were included, of which 5 were longitudinal, 4 were intervention and 1 transversal. All studies identified subclinical vitamin A deficiency in children hospitalized with pneumonia; the highest frequency of subclinical vitamin A deficiency was 93.2%. All studies evaluated showed frequencies of subclinical vitamin A deficiency >20%.

**Conclusion:** There is a high frequency of subclinical vitamin A deficiency in children with pneumonia; these data need to be further explored in terms of their associations. For this reason, new studies that evaluate this topic are of fundamental importance.

## Introduction

Respiratory diseases are among the main causes that require assistance in pediatric care units [1], and since the 1960s, they are among the top five causes of child death [2]. Among these, pneumonia is the most frequent respiratory infection and figure as a cause of morbidity and mortality in developing countries, particularly among children under 5 years of age [3, 4].

Pneumonia is an acute inflammatory disease of infectious cause that affects the spaces of the lower respiratory tract, and can be caused by viruses, bacteria, fungi, protozoa, larvae, and helminths, in addition to chemical and physical agents. Viruses and bacteria are the main etiologies of this disease [5]. Among the types of pneumonia, community-acquired pneumonia (CAP) is the most common [5] and responsible for about 80% of the deaths of children affected by respiratory tract infections [6, 7].

Estimates suggest that due to severe clinical complications, 7%–13% of all known cases of pneumonia require advanced care in a hospital setting. Not infrequently, the most severe cases evolve to death, especially in children under 1 year of age [8].

The risk factors for this condition are considered to be of socioeconomic origin (low income, parental education, access to health services) environmental (home smoking, environmental pollution close to home, lack of sanitation, humidity, mold, climate and temperature variation), nutritional and perinatal (prematurity, low and extremely low birth weight, associated morbidities during pregnancy and birth, lack of breastfeeding or early weaning) [9, 10], outdated vaccination schedule, pathogen virulence, the immune response of each exposure to pollution and environmental allergens [11–13].

From the perspective of nutritional factors, the physiological concentrations of retinoids have been implicated in organic resistance against infections. In this context, there is evidence that retinoids modulate the response of phagocytic cells and the increase in the percentage of lymphoid cells with expression of “Natural Killer” (NK) cell markers, which suggests a different performance of the various retinoids in specific cell immunity [14–16].

In the 1980s and 1990s, studies emerged that associated vitamin A deficiency and morbidity and mortality from infectious diseases, especially those in which the function of the epithelium is compromised, such as measles, diarrhea and respiratory diseases [17–19].

Vitamin A deficiency can be caused by two main factors. The first is the inadequate intake of vitamin A to satisfy organic needs, such as insufficient consumption of animal products and fruits and vegetables rich in pro-vitamin A, leading to an inefficient absorption of this micronutrient. The second is related to the synergism between infectious episodes and vitamin A deficiency [20, 21].

The association between vitamin A deficiency and morbidity has several possible explanations: vitamin A deficiency, in addition to its known consequences on vision, has an effect on the structural integrity, differentiation and maintenance of epithelial tissues (which constitute one of the first lines of non-specific defense), and immunity, among other functions [22, 23]. Vitamin A is an essential nutrient for various physiological processes related to vision, growth, cell differentiation, hematopoiesis and immune system reactivity. However, there is still no consensus in the literature regarding the effectiveness of this supplementation in the case of respiratory disorders [24, 25].

Considering that pneumonia is an important cause of illness and death in children, especially in the poor and developing regions of the planet, and that nutritional deficiencies are highly prevalent in these countries, studies that evaluate this topic are fundamental to support strategies for minimization of these health problems of great repercussion on public health, such as vitamin supplementation as a health policy.

In addition, studies with this theme are scarce, where the last studies that addressed this subject are mostly from the 1990s, with the main focus being vitamin A supplementation in children with pneumonia. Thus, further studies are needed to rekindle the discussion around the importance of the vitamin A nutritional status and hospitalization for pneumonia in children and whether there is consensus on the topic, since both hypovitaminosis A and pneumonia are morbidities of fundamental importance within the world scenario.

In this perspective, the aim of the present study was to carry out an integrative review to identify the prevalence of vitamin A deficiency in children aged 6 months to 5 years hospitalized for pneumonia.

## Methods

An integrative review was carried out, according to the following steps: establishment of the issue for review, establishment of inclusion and exclusion criteria for articles (sample selection); definition of the information to be extracted from the selected articles; analysis of results; presentation of results and discussion [26]. The guiding question for this review was “What is the frequency of vitamin A deficiency in children aged 6 months to 5 years hospitalized for pneumonia?” The procedures described below were performed by two independent researchers previously trained (ACLM and AMGS).

Primary studies with children from 6 months to 5 years of age hospitalized for pneumonia and who had measured serum retinol levels at hospital admission were included. There were no limitations on language or publication time. The search for articles was carried out in the databases PubMed, LILACS, Web of Science, Scopus and CINAHL, and in the gray literature with OpenGrey, Proquest and Google Scholar. In addition to manual search of the bibliographic references of the works included.

In the first search in the databases there were no language limitations or publication time and it took place on 7 October 2019, after which the exclusion of duplicate works was carried out. The title and summary analysis was performed using the Rayyan QCRI application [27]. When necessary, the third reviewer (IRB) resolved the conflicts. In the eligibility phase, the screened studies were read in full and the bibliographic references of the selected articles were analyzed to identify possible additional studies to the review. A new search was carried out on 12 June 2022, to update new studies, within the period from 2019 to 2022 and following all the steps of the first search.

The selected articles were analyzed when reading the full text and evaluated according to the inclusion and exclusion criteria established and that answered the research question focused on the integrative review. The following information was extracted: author, year of publication, age, city and country in which the study was conducted, mean serum retinol levels and frequency of vitamin A deficiency. The unit of measurement adopted for serum retinol levels was µmol/L, those studies that differed from this unit, were converted by the authors to unify the measure.

The assessment of methodological quality was measured using the Downs and Black (1998) checklist [28]. Twenty-seven (27) questions were analyzed corresponding to the following domains: report, external validity, bias, confounding variable and power. The score from 0 to 1 adopted for the evaluation of each item was developed by the researchers, where 1 corresponded to the item met, 0.5 to the item partially met, and 0 for items not met or unable to be determined.

## Results

**FIGURE 1:**
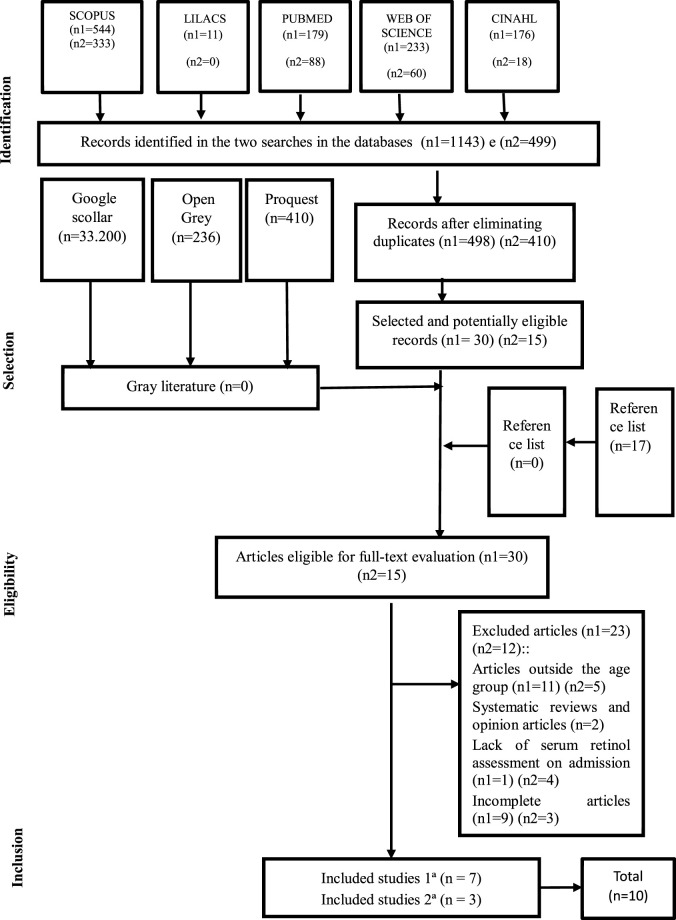
Flowchart of article selection. Natal, RN, Brazil, 2019. Adapted from PRISMA [29].

The search strategies used in the selected databases are described in Table 1. The selection of articles followed the flowchart adapted from the prism (Figure 1), for articles that were incomplete contacts were made electronically with their respective authors, but without success, remaining at the end of the first search 7 articles. In the second search, 3 more articles were included, totaling 10 full articles that met the inclusion criteria.

**TABLE 1:** Search strategy for the selected databases. Natal, RN, Brazil, 2019.

Database	Strategy	Result 1	Result 2
Scopus	(“Vitamin A″ OR “Carotenoids” OR “Retinoids” OR “Retinol”) AND (“Pneumonia” OR “Pneumonia, Bacterial” OR “Pneumonia, Pneumocystis” OR “Pneumonia, Viral”)	544	333
LILACS	(“Vitamina A″) AND (“pneumonia” OR “neumonía”)	11	0
PubMed	(“Vitamin A”) AND (“pneumonia”)	179	88
Web of science	(“Vitamin A″ OR “Carotenoids” OR “Retinoids” OR “Retinol”) AND (“Pneumonia”)	233	60
CINAHL	(“Vitamin A″ OR “Carotenoids” OR “Retinoids” OR “Retinol”) AND (“Pneumonia”)	176	18
Total		1143	499

These articles were evaluated for methodological quality [28] and had a minimum and maximum score of 13 and 22 points (Table 2), respectively, with an average of 64.4% of the checklist score.

**TABLE 2:** Methodological quality according to the Downs and Black (1998) checklist [28]. Natal, RN, Brazil, 2019.

References	1	2	3	4	5	6	7	8	9	10	11	12	13	14	15	16	17	18	19	20	21	22	23	24	25	26	27	Total
[30]	1	0	1	1	0.5	1	1	0	1	1	0	0	1	1	1	1	1	1	1	1	1	1	1	1	0	1	1	21.5
[24]	1	1	1	1	0.5	1	1	0	1	1	0	0	1	1	1	1	1	1	1	1	0	1	1	1	0	1	0	20.5
[31]	1	1	1	1	0	1	1	1	1	1	0	0	1	1	1	1	1	1	1	1	0	1	1	1	0	1	1	22.0
[25]	1	1	1	0	0	1	1	0	0	1	0	0	1	0	0	1	1	1	0	1	1	1	0	0	0	0	0	13.0
[32]	1	1	1	1	0	1	1	0	0	1	0	0	0	1	1	1	1	0	1	1	1	1	1	1	0	0	1	18.0
[33]	1	1	1	0	0	1	1	0	0	1	0	0	1	0	0	1	1	1	1	1	1	1	0	0	0	0	0	14.0
[34]	1	1	0	0	0	1	1	0	0	1	0	0	1	0	0	1	1	1	1	1	0	1	0	0	0	0	1	13.0
[35]	1	1	1	1	1	1	1	0	1	1	0	0	1	0	0	1	1	1	0	1	1	1	0	0	1	0	1	18.0
[36]	1	1	1	1	0	1	1	0	1	1	0	0	1	0	0	1	1	1	0	1	1	1	0	0	0	0	1	16.0
[37]	1	1	1	1	1	1	1	0	1	1	0	0	1	0	0	1	1	1	0	1	1	1	0	0	1	0	1	18.0

1- Hypotheses; 2- outcomes; 3- Patient characteristics; 4- interventions; 5- Confusion factors; 6- Findings of the studies; 7- Random variability; 8- Adverse effects; 9- Lost participants; 10- Confidence interval (95%); 11- Representativeness of the population of the study site; 12- Representativeness of the whole population; 13- representative place of treatment; 14- Blinding participants; 15- Blinding of the measurers; 16- Data dredging; 17- adjusted analyses; 18- Statistical tests; 19- Reliability of adhesion; 20- Accurate measurement; 21- Recruitment population of patients; 22- Patient recruitment time; 23- randomization; 24- Hidden randomization for patients and team; 25- Adjusting confusion factors; 26- Losses of patients; 27- Power of the study.

Of the 10 articles included, 5 were of the longitudinal type, 4 of interventional and 1 transversal. The studies investigated different populations, age groups, diagnostic criteria for pneumonia and vitamin A deficiency. Some studies demonstrated the child’s follow-up after hospital discharge [24, 30, 32, 33]. Table 3 shows the characterization of countries, age group, mean serum retinol level and frequency of vitamin A deficiency.

**TABLE 3:** Characteristics of the included studies. Natal, RN, Brazil, 2019.

Authors	City/Country	Age group of the sample	Study type	Serum retinol levels (mean)	Frequency of vitamin A deficiency (%)	Did children hospitalized for pneumonia have vitamin A deficiency on admission?
[30]	Maputo-Mozambique	6–72 months	Intervention	0.3 μmol/L*(IG) 0.28 μmol/L*(PG)	Subclinical deficiency: 93.2% (<0.7 μmol/L) Clinical deficiency: 68.9% (<0.35 μmol/L)	YES
[24]	Guatemala city Guatemala	3–48 months	Intervention	0.92 μmol/L(IG) 0.87 μmol/L(PG)	Vitamin A deficiency (<0.7 μmol/L): 28% in both groups	YES
[32]	Recife-Brazil	6–59 months	Intervention	0.45 μmol/L(IG) 0.38 μmol/L(PG)	Vitamin A deficiency (<0.7 μmol/L): 82% (IG) 86% (PG)	YES
[25]	Havana- Cuba	3 months–156 months	Cohort	0.77 μmol/L* (PNMC) 0.76 μmol/L* (PNMNC)	Subclinical deficiency (<0.7 μmol/L): 48% Clinical deficiency (<0.35 μmol/L): 8% Altered conjunctival cytology: 47%	YES
[31]	Recife- Brazil	6–59 months	Intervention	1.04 μmol/L(IG) 0.91 μmol/L(PG)	Vitamin A deficiency (<0.7 μmol/L): 37.1% (IG) 38.3% (PG)	YES
[33]	Santo André- São Paulo- Brazil	6 months–59 months	Cohort	1.4 μmol/L(AP) 1.7 μmol/L(RP)	Inadequate serum retinol levels (<1.05 μmol/L): AP: 32.5% RP: 17.5%	YES
[34]	Jiangsu –China	6–36 months	Control case	1.3 μmol/L*	Vitamin A deficiency (<0.7 μmol/L): 46.59%	YES
[35]	Beijing, China	6–204 months	Cross-sectional	1.08 µmol/L* (RRTI) 0.98 µmol/L* (RTI) 0.94 µmol/L* (RRTI and RTI)	Subclinical deficiency (<0.7 μmol/L): 38.56% Clinical deficiency (<0.35 μmol/L): 9.35%	YES
[36]	Ile-Ife –Nigeria	2–168 months	Control case	0.97 µmol/L*	Vitamin A deficiency (<0.7 μmol/L):15.3%	YES
[37]	Chongqing, China	6–216 months	Control case	0.59 µmol/L* (GMPP) 0.38 µmol/L* (RMPP)	Vitamin A deficiency (<0.7 μmol/L): 31.75% (GMPP) 68.75% (RMPP)	YES

*Measures converted by the authors; IG, intervention group; PG, placebo group; PNMC, complicated pneumonia; PNMNC, uncomplicated pneumonia; AP, acute phase; RP, resolution phase; RRTI, recurrent respiratory tract infections; RTI, respiratory tract infections; GMPP, General M. pneumoniae pneumonia; RMPP, Refractory M. pneumoniae pneumonia.

Table 3 shows that the classification of vitamin A deficiency differed in some studies, Julien et al. [30], Moreira et al. [25] and Wang et al. [35], categorized subclinical and clinical vitamin A deficiency serum retinol levels below <0.7 μmol/L and 0.35 μmol/L, respectively. Kjolhede et al. [24], Nacul et al. [31, 32], Zuo et al. [34], Kuti et al. [36], and Li et al. [37], classified vitamin A deficiency values below <0.7 μmol/L without categorizing it into subclinical or clinical deficiency, as well as Silva et al. [33], who used another cutoff point to classify the inadequate serum retinol level (<1.05 μmol/L). The lowest and highest mean of serum retinol presented were in the study by Julien et al. [30] in the placebo group (0.28 μmol/L) and in the study by Silva et al. [33] in the acute phase of infection (1.4 μmol/L), respectively.

Julien et al. [30] were also the ones who identified the highest frequency 93.2% and 68.9% of subclinical and clinical disabilities, respectively; Moreira et al. [25] had the lowest frequency considering clinical disability (8%). In addition to the assessment of serum retinol levels, Moreira et al. [25] also detected altered conjunctival cytology in 47% of the children evaluated. It is also noteworthy that the mean plasma retinol levels were significantly higher in the pneumonia resolution phase (1.7 μmol/L), when compared to the acute phase of infection (1.4 μmol/L) in the study by Silva et al. [33].

## Discussion

This integrative review aimed to identify the frequency of vitamin A deficiency in children hospitalized for pneumonia at the time of hospital admission, since this vitamin is essential to the immune system and that negatively repercussions when there is clinical or subclinical deficiency [38, 39].

All included studies have passed by methodological quality and presented good results. The main limitations identified in most studies were the lack of assessment and/or correction of bias. [25, 30–34] of the 4 intervention studies, only Nacul et al. [31] identified the adverse effects of intervention. As well as, none of the studies assessed sample representativeness with regard to the source or target population.

The age group of evaluation of the studies presented differed, ranging from 2 months to 18 years, indicating a great heterogeneity of the population evaluated; however, this fact did not prejudice the evaluation data, since the classification values of serum retinol levels is equal for the various age groups. In relation to the place of study, all were carried out in developing countries that historically present higher prevalence of deficiency and infectious diseases corroborating with data presented by organs as WHO [40] and Ministry of Health of Brazil [41], in addition to others [42, 43].

As for the classification of vitamin A deficiency, despite differing between the studies analyzed in relation to the cutoff point and units used, all presented high frequencies of subclinical and/or clinical deficiency in children hospitalized for pneumonia. The highest frequency of subclinical and clinical deficiency of vitamin A was 93.2% and 68.9%, respectively [30] and the lowest values [25] were with 8% of children hospitalized by pneumonia presenting vitamin A clinical deficiency. With the lowest average serum retinol presented by Julien et al. [30], (0.28 μmol/L) and the largest by Silva et al. [33], (1.4 μmol/L).

Considering as subclinical deficiency of vitamin A levels below 0.7 μmol/L [37], all studies evaluated presented prevalence> 20% of this deficiency, except for the study by Bankole et al. [36], which showed 15.3%. As well as the study by Silva et al. [33], which used another cutoff point (lower levels 1.05 μmol/L) to consider inadequate levels of vitamin A; however, for WHO [44] values between 0.7 and 1.04 μmol/L are considered acceptable.

This hypovitaminosis can lead to a negative repercussion in the health of the child and appears as an important factor in determining the morbidity and mortality of the child population. In addition to the deficit in immunity and respiratory epithelium, it may lead to a scaly metaplasia with consequent loss of defense mechanisms against the invasion of microorganisms, generating the trigger of obstructive phenomena caused by increased bronchial reactivity [45].

It is also highlighted that Silva et al. [33], concluded that plasma retinol levels are significantly higher in the resolution phase of pneumonia compared to acute phase. Acute respiratory infection can attend with plasma levels of smaller retinol by several factors, among them: greater consumption with the objective of recovering the damaged tracheobronchial epithelium; decreased ingestion and absorption; Deviation from protein synthesis, increased antioxidant consumption due to oxidative stress generated by inflammation and infection; and increase urinary excretion during the acute phase of infection [46–49].

Under normal conditions, the retinol stored in the liver binds to the retinol carrier protein; this protein joins the transthyretin and carries out the transport of retinol to peripheral tissues. Due to the decrease in hepatic synthesis of these proteins during the systemic inflammatory response, there is progressive reduction of serum retinol during infection. The recovery of the infection is likely to be associated with the progressive increase in the retinol carrier protein and serum retinol levels [48–50].

At the same time at the serum retinol level, Moreira et al. [25], also analyzed the conjunctival cytology altered in 47% of the children and found a significant association between the altered cytology and the severity of pneumonia, where cases of altered conjunctival cytology had 2.2 greater risk of presenting severe pneumonia. Conjunctival cytology is used by its ability to assess vitamin A sufficiency to maintain the integrity of the epithelium. In people with vitamin A deficiency, after mega doses of retinol the conjunctiva takes about three to 8 weeks to redefine its normal cytological pattern, indicating that vitamin A deficiency is related to previous periods and not by possible transitional reduction of phase protein acute [51].

The study by Li et al. [37], identified lower serum retinol levels in children with refractory M. pneumoniae pneumonia, decreased vitamin A during M. Pneumoniae infection can worsen lung lesions which may contribute to the development of refractory M. Pneumoniae pneumonia.

According to the World Health Organization [44], the prevalence of serum levels of retinol <0.70 μmol/L in 2%–10% of the child population of 6–71 months of age indicates a light public health problem; 10%–20%, moderate problem and >20%, severe. In this way, the data presented in these studies are worrying, since the frequency of subclinical deficiency was above 20% for most of the studies evaluated, reaffirming that this nutritional deficiency remains as a public health problem all over the world, especially in developing countries, since the included studies reported the occurrence of this problem in Mozambique, Guatemala, Brazil, Cuba, China and Nigeria.

Vitamin A deficiency is a deficit disease that occurs mainly between the low socioeconomic groups that feed poorly and live in unsatisfactory health conditions [38]. The VAD is disseminated in Southeast Asia, in the Middle East, Africa and Central and South America, especially in children, and is associated with general malnutrition, although there is a trend of decline in the World Prevalence of VAD, significant reductions in 1991–2013 in East and Southeast Oceania, from 42% to 6%, and in Latin America and Caribbean of 21%–11% [38–52]. The highest rates of vitamin A deficiency are in sub-Saharan Africa and South Asia, at 48% and 44%, respectively [53].

Regions that obtained a reduction in the prevalence of VAD were often attributed to government actions, in particular the mass management of high doses of vitamin A with children in the last 20 years [54]. The periodic supplementation of high doses of vitamin A is a proven intervention of low cost, which reduces mortality by all causes by 12%–24% and therefore is an essential program in support of strategies to reduce infant mortality [53].

In view of the positive impact of supplementation, the World Health Organization recommends the administration of vitamin A supplements in children aged 6–59 months, and that this prophylactic supplementation should be part of a set of strategies for improving the intake of this nutrient, therefore, associated with diversification of diet [55].

Inadequate feeding encompasses deficiency in food intake vitamin A source, as well as insufficient consumption of food containing important nutrients for its bio utilization. Food consumption is conditioned by cultural factors, such as food habits, individual and family preferences, and by socioeconomic factors that affect the ability to choose and purchase these foods [56, 57].

Therefore, vitamin A supplementation is a short-term measure, effective to combat VAD. Nutrition education can be useful in the long run as a complement to supplementation and fortification of food [20].

The incidence of pneumonia among children under 5 years in developing countries is five times higher than in developed countries [58], and forecasts show that 6.3 million children under 5 years old may die of pneumonia between 2020 and 2030, according to Current trends [59]. The implementation of a vitamin A supplementation policy in poor and developing countries may be crucial to reducing pneumonia morbidity and mortality, minimizing negative impacts in these regions of high social inequality and shortage of human and financial resources [9].

It is worth mentioning some limitations of this review, such as the methodological differences for estimation of vitamin A levels in the included studies, the small number of included studies and old publications. This fact reiterates the need for new primary studies with this theme, since vitamin A deficiency and pneumonia are public health problems and that despite efforts to reduce child health impacts the two pathologies are frequent and can cause permanent damage to children development. Despite limitations, this integrative review was carried out with strict methodology by independent authors.

### Conclusion

This review evidenced high frequency of subclinical vitamin A deficiency in children hospitalized for pneumonia. However, more studies are needed to verify this association. Most of the studies analyzed suggest that prophylactic supplementation is essential mainly in developing countries to reduce child morbidity and mortality. In addition, adjuvant vitamin A supplementation in children with pneumonia should be more investigated for standardization of guidelines and consensus in order to create protocols and strategies to minimize the alarming numbers of these pathologies that are considered avoidable, but still very frequent in all parts of the world.
